# Capability of coupled CdSe/TiO_2_ heterogeneous structure for photocatalytic degradation and photoconductivity

**DOI:** 10.1186/1556-276X-9-636

**Published:** 2014-11-26

**Authors:** Miao Zhang, Yanyan Xu, Jianguo Lv, Lei Yang, Xishun Jiang, Gang He, Xueping Song, Zhaoqi Sun

**Affiliations:** 1 School of Physics and Material Science, Anhui University, 111 Jiulong Rd., Hefei 230601, Peoples’ Republic of China; 2 School of Electronic and Information Engineering, Hefei Normal University, 373 Huangshan Rd., Hefei 230601, Peoples’ Republic of China; 3 Army Officer Academy, 451 Huangshan Rd., Hefei 230031, Peoples’ Republic of China; 4 School of Electronic and Electrical Engineering, Chuzhou University, 1528 Fengle Rd., Chuzhou 239000, Peoples’ Republic of China

**Keywords:** TiO_2_ nanotubes, CdSe quantum dots, Cyclic voltammetry, Photocatalysis

## Abstract

Highly ordered TiO_2_ nanotube arrays (TiO_2_-NTAs), with a uniform tube size on titanium substrate, were obtained by means of reoxidation and annealing. A composite structure, CdSe quantum dots@TiO_2_ nanotube arrays (CdSe QDs@TiO_2_-NTAs), was fabricated by assembling CdSe quantum dots into TiO_2_-NTAs via cyclic voltammetry electrochemical deposition. The X-ray diffractometer (XRD), field-emission scanning electron microscope (SEM), and transmission electron microscope (TEM) were carried out for the determination of the composition and structure of the tubular layers. Optical properties were investigated by ultraviolet-visible spectrophotometer (UV-Vis). Photocurrent response under visible light illumination and photocatalytic activity of samples by degradation of methyl orange were measured. The results demonstrated that the photo absorption of the composite film shifted to the visible region, and the photocurrent intensity was greatly enhanced due to the assembly of CdSe QDs. Especially, photocurrent achieved a maximum of 1.853 μA/cm^2^ after five voltammetry cycles of all samples. After irradiation under ultra violet-visible light for 2 h, the degradation rate of composition to methyl orange (MO) reached 88.20%, demonstrating that the CdSe QDs@TiO_2_-NTAs exhibited higher photocatalytic activity.

## Background

Very recently, highly ordered TiO_2_ nanotube arrays (TiO_2_-NTAs), which were synthesized by anodic oxidation on titanium substrate, had attracted great attention in recent years for solar cells [1], photocatalysis [2], water photoelectrolysis [3], and so on. However, due to its wide band gap of TiO_2_ (*E*_g_ *=* 3.2 eV [4]), only ultraviolet (UV) region with the wavelength below 390 nm of the solar spectrum can be utilized, which prevents its potential application. Therefore, much effort has been dedicated to expanding the photocatalytic function of the TiO_2_-NTAs to the visible light region [5]. Quantum dot-sensitized solar cells (QDSSCs) are renowned energy devices known in the past decade for their distinct qualities, including absorb light in the visible region, simplicity in fabrication, tunable band gaps [6], and low cost. Of particular interest are CdX (X = S [7,8], Se [9], and Te [10]) QDs, which have small and size-dependent band gaps and thus provide new opportunities for harvesting light energy in the visible and infrared regions of solar light [11-13].

It has been noticed that TiO_2_-NTAs anodized only after chemical polishing were of various lengths and able to show the blocked nanotube mouth, which prevents the continued investigation, such as sensitization by QDs. To solve this issue, a simple method to obtain uniform and highly ordered TiO_2_-NTAs via reoxidation has been developed in the present study, which similar with our previous work [14]. Followed by annealing process, CdSe QDs were assembled onto the crystallized TiO_2_-NTAs by cyclic voltammetry at different cycles in a conventional three-electrode cell using an electrochemical workstation. The microstructure, composition, optical activity, and photocatalytic effect of CdSe QDs@TiO_2_-NTAs were investigated systematically.

## Methods

### Preparation of TiO_2_ nanotubes (NTAs) and decoration

Ti foils (99.6% purity, 0.2 mm) were divided through wire-electrode cutting into 1 × 2 cm^2^, then cleaned ultrasonically in acetone, alcohol, and de-ionized (DI) water for 5 min in turn. Chemical polishing was adopted to remove impurities adhered to the surface of Ti foils. The polishing solution consists of hydrofluoric acid (HF, AR), nitric acid (HNO_3_, AR), and de-ionized (DI) water, with the volume ratio of 1:1:2. At last, cleaned Ti foils were dried in nitrogen stream. The TiO_2_-NTAs were synthesized in a two-electrode cell containing a cathode of platinum foil at 60 V with weak magnetic stirring. The electrolyte was composed of 0.3 wt.% of NH_4_F (96% purity, AR), glycol (>99% purity, AR), and 2 vol.% of DI water, similar to previous investigation described by Grimes et al. [1]. After 4 h of anodization, the samples were sonicated in DI water and carbinol (>99.5% purity, AR), respectively, and then dried by a drying oven to take off the first TiO_2_-NTAs films from substrate. Followed by cleaning, the reoxidation was carried out at ‘bowl-like’ Ti foil substrate at the same voltage and lasted for the same time. The above process has always been under room temperature. Before the CdSe sensitization, a subsequent heating at 350°C for 2 h with a temperature ramp rate of 2°C ⋅ min^-1^ in air was applied to achieve the crystallized TiO_2_-NTAs.

### Synthesis of CdSe@TNAs

CdSe QDs were fabricated onto the TiO_2_-NTAs by cyclic voltammetry. Electrodeposition of CdSe QDs@TiO_2_-NTAs was performed in a conventional three-electrode cell using a Chi660D electrochemical workstation (Cheng Hua Instruments, Inc., Shanghai, China). The working electrode, the counter electrode, and the reference electrode were TiO_2_-NTAs after calcined at 350°C, a Pt foil and a saturated calomel electrode (SCE, filling solution is KCl of 3.5 mol/L), respectively. The electrolyte solution was prepared by mixing 0.25 M CdSO_4_, 0.25 M HNO_3_, and 0.015 M Na_2_SeO_3_ at ambient temperature using cyclic voltammetry technique, sweeping the potential between 0 and -1 V (vs. SCE) at a sweep rate of 0.1 V/S for different number of cycles (marked as ‘r’ in the Figures). CdSO_4_, HNO_3_, and Na_2_SeO_3_ were obtained from Sinopharm Chemical Reagent Co., Ltd., Shanghai, China. All reagents used were analytical grade. After electrochemical deposition, the samples were thoroughly rinsed by DI water and annealed at 350°C in vacuum atmosphere for 2 h. The samples were noted as r0, r3, r5, r7, and r9 according to the cycles of cyclic voltammetry (0, 3, 5, 7, and 9 times, respectively).

### Characterizations and measurements

The surface morphology and thickness of TiO_2_-NTAs films were characterized by field-emission scanning electron microscope (SEM, Hitachi, S4800; Hitachi Ltd., Chiyoda-ku, Japan) and high-resolution transmission electron microscopy (HRTEM, JEM-2100; JEOL Ltd., Akishima-shi, Japan). Microstructures of TiO_2_-NTAs were conducted by X-ray diffractometer (XRD, MAC, M18XHF, Shimadzu, Kyoto, Japan) employing CuKα radiation. The absorption spectra of samples were recorded by an ultraviolet-visible (UV-Vis) spectrophotometer (UV-2550, Shimadzu, Kyoto, Japan) within the wavelength range of 300 to 900 nm.

The photoresponse characteristics of CdSe QDs@TiO_2_-NTAs heterostructures were evaluated by electrochemical workstation (CHI600D) in a photoelectron chemical cell under intermittent visible light illumination (100 mW/cm^2^ AM 1.5) with 0 V bias potential (vs. SCE) in 0.5 M Na_2_SO_4_ aqueous solution. Photocurrents were investigated in a three-electrode system under visible light illumination.

At last, the CdSe QDs sensitized TiO_2_-NTAs were employed as photocatalyst for the degradation of methyl orange (MO) and compared with pure TiO_2_-NTAs under UV lamp irradiation. The experiment was performed in a glass container. The samples were immersed in 10 mL ⋅ 15 ppm MO solutions and were irradiated with a 36 W high-pressure mercury lamp, which emits visible light of 404.7, 435.8, 546.1, 577.0, and 579.0 nm, and ultraviolet light of 365 nm. The distance between the sample and the high-pressure mercury lamp was 5.0 cm. The transmittance of the MO solution was measured at intervals of 10 min, and the total irradiation time is 120 min.

## Results and discussion

### Structural study

**Figure 1:**
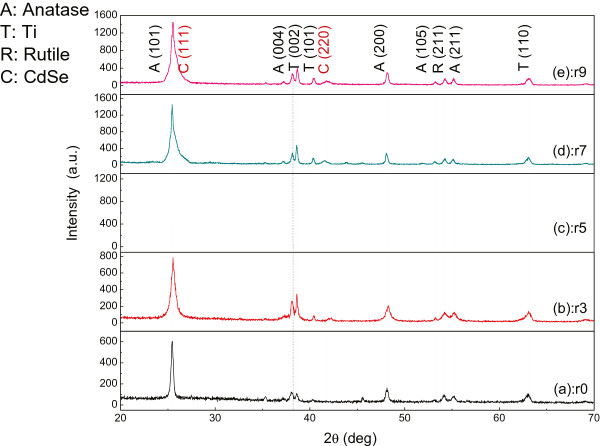
**XRD patterns of CdSe QDs@TiO**_**2**_**-NTAs heterogeneous structure prepared by voltammetry electrochemical deposition in different cycles.** (a) 0, (b) 3, (c) 5, (d) 7, and (e) 9 times.

Figure 1 shows the crystal structure of the TiO_2_-NTAs before and after modification with CdSe which prepared through different cycles. The bare TiO_2_-NTAs show only peaks corresponding to the anatase TiO_2_ (marked by ‘A’) and rutile TiO_2_ (marked by ‘R’). The patterns of CdSe nanoparticles anchored TiO_2_-NTAs show only a broader peak at 25.35° for the overlapped patterns of (1 1 1) planes of zinc blende CdSe (approximately 25.35°) and (1 0 1) planes of anatase TiO_2_ (approximately 25.28°). The peaks located at approximately 42.01° corresponded to the (2 2 0) crystalline planes of cubic CdSe (marked by ‘C’). Nevertheless, the broadened XRD peak indicates the dispersion of very small CdSe nanocrystallites. It is worth noting that a well-crystalline material is good for photovoltaic application owing to its high charge transport properties and low recombination losses [15]. Continuing increasing cycling times, the diffraction peaks of CdSe intensity strengthened accordingly. It was indirectly proven that the loaded CdSe amount increased as the growing cycles increased.

### Morphological characterization

**Figure 2:**
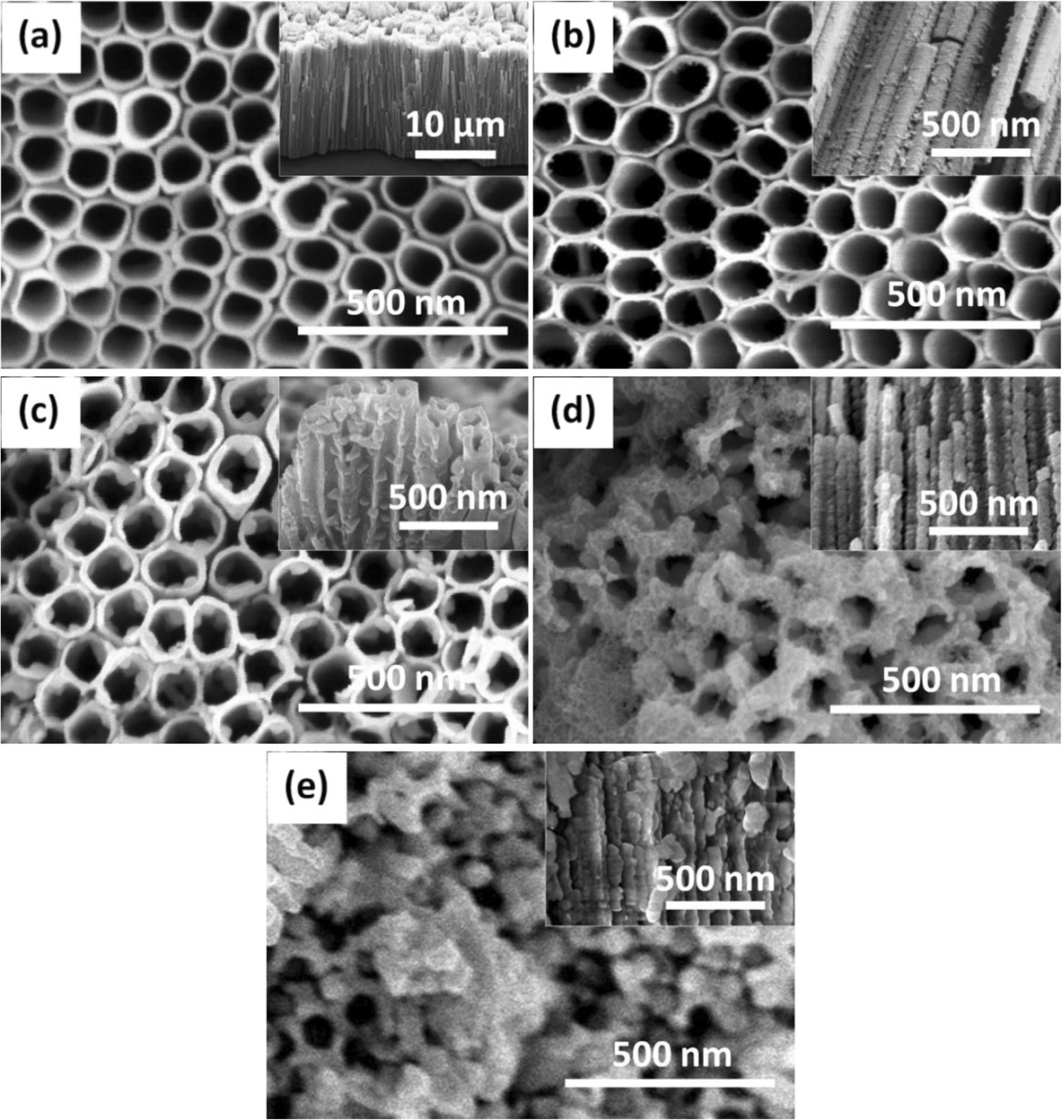
Top view image (a) and different cycles of cyclic voltammetry deposition (b-e).

The SEM images of TiO_2_-NTAs before and after modification with CdSe are shown in Figure 2. Uniform and well-aligned nanotubes vertically oriented from the Ti foil substrate can be seen from Figure 2a. The length of TiO_2_-TNTs is over 10 μm long with an outer average diameter of about 120 nm.TiO_2_-NTAs modified by CdSe QDs deposited by cyclic voltammetry are shown in Figure 2a,b,c,d,e. Figure 2a shows the blank tube with length around 10 μm. From Figure 2b, it can be seen that a small amount CdSe QDs have deposited at the top and wall (show in inset) of TiO_2_-NTAs after three cycles. After five cycles, the number of CdSe QDs increases obviously, with good dispersion onto TiO_2_-NTAs, as shown in Figure 2c. After seven cycles, large amount of CdSe QDs agglomerate into larger particles in the inner and outer wall of nanotubes. Most of the TiO_2_ nanotubes are blocked by CdSe QDs, which can be seen in Figure 2d. After nine cycles, all the TiO_2_-NTAs were completely blocked by CdSe QDs, and open mouth nanotubes had not been observed, as shown in Figure 2e. The inset is a cross-section view.

**Figure 3:**
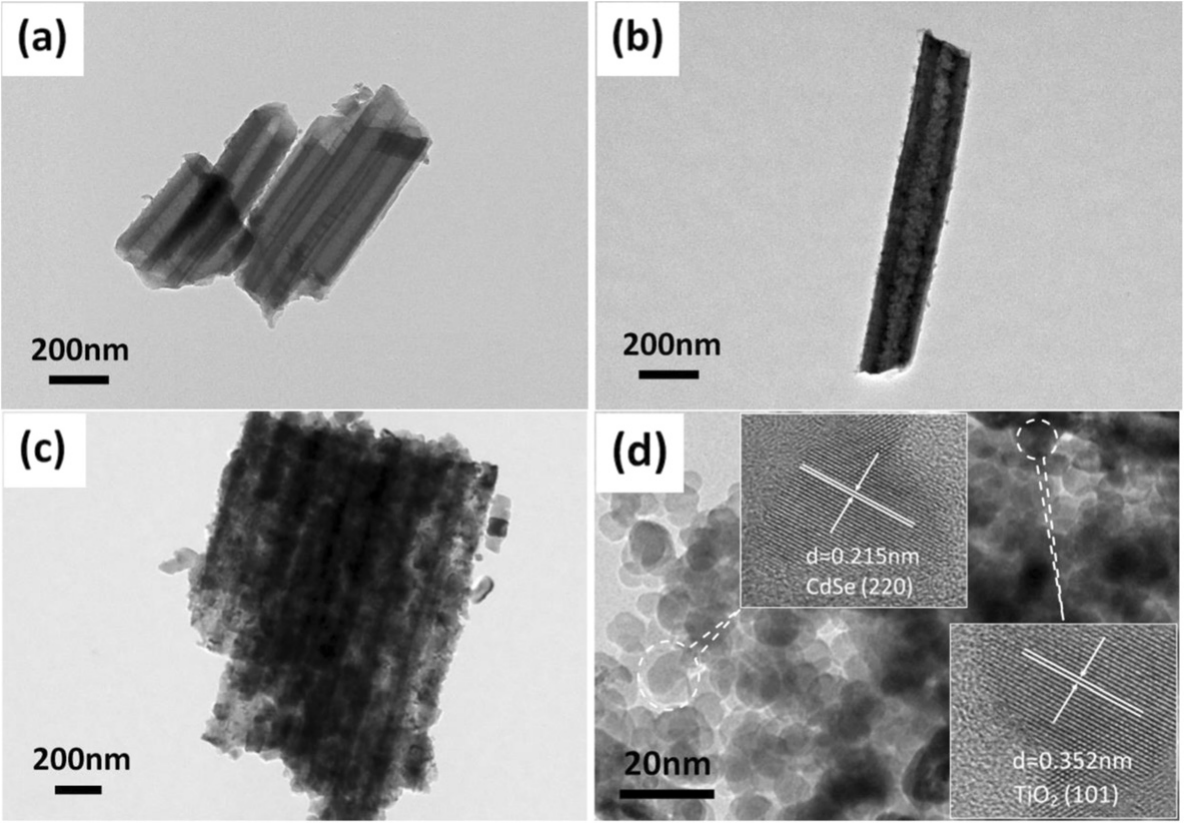
**TEM images of TiO**_**2**_**-NTAs and TEM and HRTEM images of CdSe QDs@TiO**_**2**_**-NTAs. (a)** TEM images of blank TiO2-NTAs, and **(b-d)** TEM and HRTEM images of CdSe QDs@TiO2-NTAs heterosturuectures prepared by voltammetry electrochemical deposition.

Typical TEM images of blank TiO_2_-NTAs were shown in Figure 3a. These nanotubes have uniform size with the average diameters of more than 100 nm, which is in agreement with SEM images. After CdSe quantum dots were sensitized, single and tens of TiO_2_ nanotube can be observed in Figure 3b,c. Compared with blank TiO_2_-NTAs, it can be seen that many CdSe QDs in a form of very tiny particles have been successfully embedded into the external and internal walls of nanotubes uniformly. The products were further characterized by HRTEM, shown in Figure 3d. The lattice spacing measured for these crystalline plane are 0.352 and 0.215 nm, corresponding to the (1 0 1) plane of anatase TiO_2_ and the (2 2 0) plane of cubic CdSe, respectively, which are marked by dashed ellipses. These results are in accordance with the XRD patterns.

### Optical characterization

**Figure 4:**
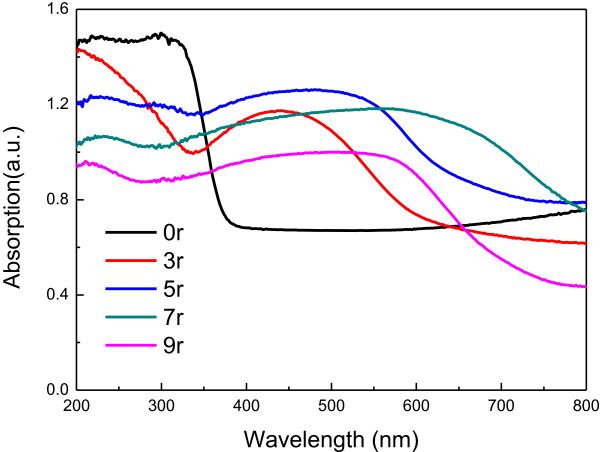
**UV absorption spectrum of the prepared TiO**_**2**_**-TNAs deposited by different amount of CdSe.**

The UV-Vis diffuse reflection absorbance spectra of TiO_2_-NTAs and the corresponding QDs-sensitized NTAs are displayed in Figure 4. As we know, pure TiO_2_-NTAs have an absorption in the UV light region and exhibit the fundamental absorption edge corresponding to the band gap energy of 3.2 eV. As shown, absorption edge for CdSe QDs@TiO_2_-NTAs thin films shifts to longer wavelength, indicating that the band gap for thin films declines with different amount of CdSe. Comparatively, after deposited with CdSe, the heterostructures exhibited higher light absorption in visible light regions. These results indicate that the deposition of CdSe extended the absorption of the TiO_2_-NTAs into the visible light region. The ordered TiO_2_-NTAs have interior surfaces on which CdSe nanoparticles can be deposited, resulting in an enhancing absorption capacity in the visible light region while collecting and transmitting electrons through the TiO_2_-NTAs. However, the aggregation of CdSe nanoparticles would decrease surfaces exposed in visible light and lead to reduced visible absorption. For this reason, it can be seen that the TiO_2_-NTAs are covered with CdSe QDs completely after nine cycles in Figure 2e, which leads to the reduction of the absorption area and the decrease in absorbance.

**Figure 5:**
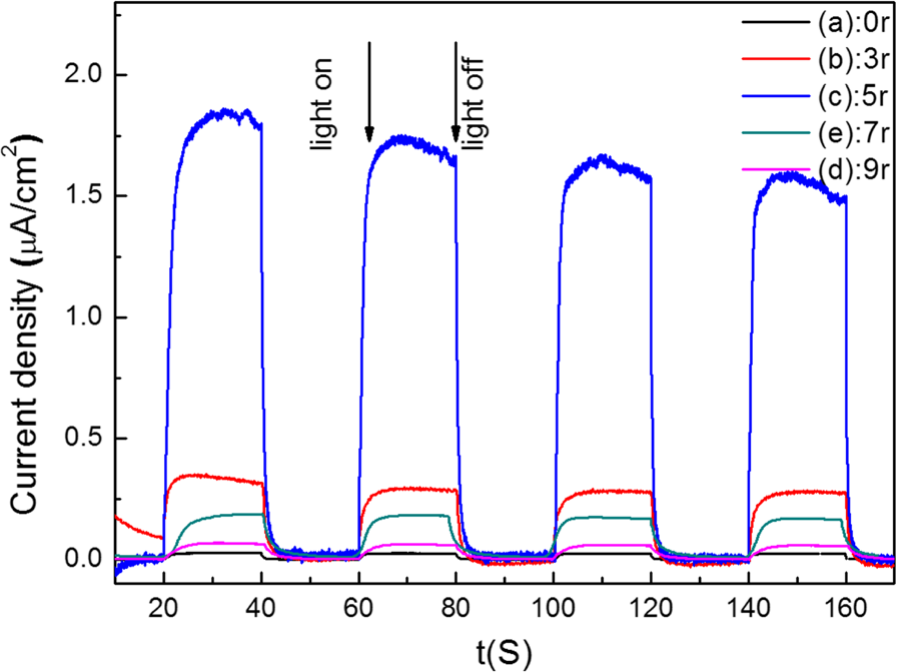
**Photocurrent response (I-t curve) of CdSe QDs@TiO**_**2**_**-NTAs in 0.5 M Na**_**2**_**SO**_**4**_**aqueous solution.**

As well known, the photocurrent response has been demonstrated to be a useful technique for evaluating the separation efficiency of photogenerated electron-hole pairs [16]. The photoresponse characteristics of CdSe QDs@TiO_2_-NTAs heterostructures were evaluated in a three-electrode system under visible light illumination with 0 V bias potential (vs. SCE). The photocurrent-time (I-t) profiles with zero bias electrode potential are shown in Figure 5.

The CdSe QDs@TiO_2_-NTAs heterostructures show evident lager photocurrent under visible light irradiation than bare TiO_2_-NTAs. This is because that CdSe is a narrowband gap semiconductor, which greatly improved the production and separation of photoinduced electrons and holes. The excited electrons in the conduction band of CdSe could be easily transferred to the conduction band of TiO_2_ and further to photocurrent collector (Ti substrate) through the highly ordered TiO_2_-NTAs structure with well crystalline nature [17]. Meanwhile, the photoexcited holes still stayed in the valence band of CdSe and were further transferred to the electrolyte, which benefit to restraining the recombination of photogenerated electrons and holes. Moreover, the reproducible and stable photoresponses are attributed to the existence of a good CdSe-nanotube interface that allows efficient electron injection from CdSe to TiO_2_-NTAs [15]. The CdSe QDs@TiO_2_-NTAs sample prepared after five cycles showed a maximum photocurrent value of 1.853 μA/cm^2^. However, when the cycles further increased to 7 times and 9 times, the photocurrent of samples exhibited obviously decreased. It is because the CdSe nanoparticles became aggregated to form nanoclusters as the cycles increased, as shown in the SEM results. For the aggregated CdSe, particles may serve as the recombination centers of photoinduced electron-hole pairs at this circumstance and cannot inject electrons into the TiO_2_-NTAs network as effectively as smaller amount of CdSe nanoparticles, which greatly hindered the separation efficiency of the excited electron-hole pairs [18,19].

Especially, photocurrent achieved a maximum of 1.853 μA/cm^2^ after five voltammetry cycles of all samples.

**Figure 6:**
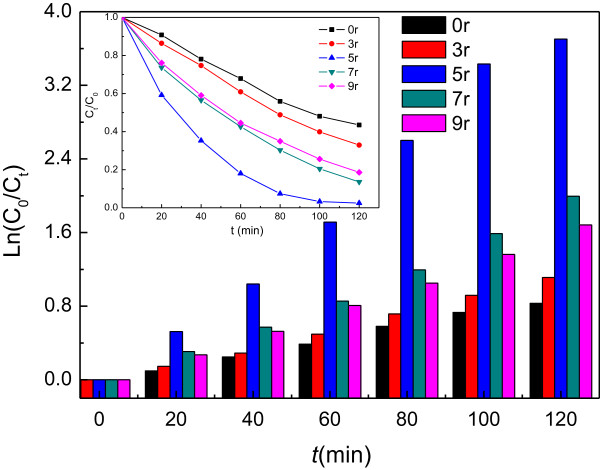
**Photodegradation curves of methyl orange (MO) using CdSe@TiO**_**2**_**-NTAs as photocatalysts.**

The photocatalytic performances of CdSe@TiO_2_-NTAs for MO degradation were shown in Figure 6 and Table 1. The degradation efficiency of MO after UV irradiation for 120 min can be described by the Lambert-Beer Law and the Langmuir-Hinshelwood model [20], *viz* Equations ([1]) and ([2])

**Table 1:** **Degradation efficiency of CdSe@TiO**_**2**_**-NTAs thin films prepared at different cycle after UV irradiation for 2 h**

**Cyclic time**	**0**	**r3**	**r5**	**r7**	**r9**
Degradation efficiency (%)	61.28	67.64	88.20	86.39	81.42

(1) $$\eta \left(\%\right)=\frac{C_{0}-C_{t}}{C_{0}}=\frac{A_{0}-A_{t}}{A_{0}}\times 100$$

(2) $$\ln\left(C_{0}/C_{t}\right)=K_{\alpha }t$$

where *C*_0_ represents the initial concentration, *C*_*t*_ represents the concentration after *t* min reaction; *A*_0_ represents the initial absorbance, and *A*_*t*_ represents the absorbance after *t* min reaction of the MO at the characteristic absorption wavelength of 464 nm. *k*_*α*_ is the apparent first-order rate constant.

It can be seen that all the CdSe@TiO_2_-NTAs samples exhibit higher MO photodegradation activity than bare TiO_2_-NTAs. This accounts for visible light photocatalytic activation of CdSe QDs. It is shown in Table 1 that the highest efficiency (88.20%) is obtained by r5 after being irradiated for 2 h. Whereas with the cyclic increased continuously, the efficiency decreased. It is widely accepted that a necessary step for semiconductor photocatalytic reaction is the generation and separation of electron-hole pairs [21]. The photocatalytic degradation of organic pollutants under light irradiation is mainly due to some active species, such as holes, $H_{2}O_{2},O_{2}^{-},OH$ and so on. When illuminated by visible light, electrons (e^-^) and holes (h^+^) were excited from CdSe because of its narrow band gap (1.75 eV).

(3) $$\text{CdSe}+h\nu \to \text{CdSe}\left(e^{-}\right)+\text{CdSe}\left(h^{+}\right)$$

The coupling of the semiconductors has a beneficial role in improving charge separation [22]. The excited electrons can transfer quickly from the CdSe conductive band to TiO_2_ conductive band, and holes accumulate in the valence band of CdSe, because conductive band (CB) of CdSe is more negative than that of TiO_2_, which prevent electron-hole recombination [23,24].

(4) $$\text{CdSe}\left(e^{-}\right)+{TiO}_{2}\to \text{CdSe}+{TiO}_{2}\left(e^{-}\right)$$

Oxygen is reduced as an electron acceptor to superoxide, and this leads to the production of hydroxyl radicals (∙OH). The formed radicals have a powerful oxidation ability to degrade organic dye. The reactions are summarized as below:

(5) $${TiO}_{2}\left(e^{-}\right)+O_{2}\to {TiO}_{2}+\cdot O_{2}^{-}$$

(6) $$\cdot O_{2}^{-}+H^{+}\to {HO}_{2}$$

(7) $${HO}_{2}\cdot +\cdot O_{2}^{-}+H^{+}\to H_{2}O_{2}+O_{2}$$

(8) $$\begin{array}{c} H_{2}O_{2}+\cdot O_{2}^{-}\to \cdot OH+{OH}^{-}+O_{2}\;\mathrm{or}\;H_{2}O_{2}\\ \;+e^{-}\to {OH}^{-}+\cdot OH \end{array}$$

(9) OH+dye→oxidationproducts

Meanwhile, the positive charged hole (h^+^) will react with OH^-^ or H_2_O adhering to the surfaces of thin films to generate ∙OH [25].

(10) $$\text{CdSe}\left(h^{+}\right)+H_{2}O\to H^{+}+\text{CdSe}+\cdot OH$$

As its morphology shown in Figure 3, the sample of 5r has open nanotube top compared with other specimen. This morphology meant once photons entered into the nanotube, it can be multiple scattered with CdSe quantum dots and TiO_2_-NTAs, that is, the sample of 5r can utilize the photons more efficiently, especially the visible light. This conclusion can be confirmed by the UV-Vis diffuse reflection absorbance spectra (Figure 4) and photocurrent-time profiles (Figure 5). On the other hand, quantum size effect of CdSe QDs plays a crucial role in enhancing photocatalytic activities of CdSe QDs@TiO_2_-NTAs, which can be verified by UV absorption spectrum. Meanwhile, large grain sizes of bulk CdSe will lead to deficient contact between CdSe and TiO_2_-NTAs and weak interactions. These results are in accordance to the SEM results.

## Conclusions

In summary, the uniform and highly ordered TiO_2_-NTAs films were obtained via reoxidation. The phase of TiO_2_-NTAs transforms to anatase phase under 350°C; it is shown that the CdSe covered both inner and outer wall of TiO_2_-NTAs efficiently through five cycles voltammetry electrochemical deposition. Size and distribution of CdSe nanoparticles were controlled by changing the electrodeposition cycles. Compared with the bare annealed TiO_2_-NTAs, the photo absorption of the composite film shifted to the visible region around 500 to 700 nm with the increase of the number of quantum dots, and the photocurrent intensity was greatly enhanced due to the assembly of CdSe QDs. Especially, photocurrent achieved a maximum of 1.853 μA/cm^2^ after five voltammetry cycles. Besides, the degradation rate of MO still reached their maximum value of 88.20% under UV lamp irradiation for 2 h. The enhanced ability makes this type of CdSe QDs@TiO_2_-NTAs promising applications in photo electrode for solar cells and photocatalyst candidate.

## Competing interests

The authors declare that they have no competing interests.

## Authors’ contributions

ZS and GH designed the experiments. MZ, LY, and JL carried out the experiments. MZ wrote the paper. XS and YX analyzed the results and participated in the revision of the manuscript. ZS and XJ proofread the manuscript and corrected the English. All authors read and approved the final manuscript.
